# Comparison of SF-36 and RAND-36 in Cardiovascular Diseases: A Reliability Study

**DOI:** 10.3390/jcm13206106

**Published:** 2024-10-13

**Authors:** Estrella García-Sánchez, Mirian Santamaría-Peláez, Eva Benito Figuerola, María José Carballo García, Miguel Chico Hernando, Juan Marcos García García, Jerónimo J. González-Bernal, Josefa González-Santos

**Affiliations:** 1 Cardiac Rehabilitation Unit, Rehabilitation Service, University Hospital of Burgos, 09006 Burgos, Spain; esgarciasanchez@saludcastillayleon.es (E.G.-S.); ebenitof@saludcastillayleon.es (E.B.F.); mjcarballo@saludcastillayleon.es (M.J.C.G.); mchicoh@saludcastillayleon.es (M.C.H.); jgarciagar@saludcastillayleon.es (J.M.G.G.); 2 Department of Health Sciences, Faculty of Health Sciences, University of Burgos, Paseo de los Comendadores s/n, 09001 Burgos, Spain; jejavier@ubu.es (J.J.G.-B.); mjgonzalez@ubu.es (J.G.-S.)

**Keywords:** cardiovascular disease, health-related quality of life, SF-36, RAND-36, reliability

## Abstract

**Background/Objectives**: Cardiovascular diseases are one of the leading causes of morbidity and mortality worldwide. Health-related quality of life is crucial to assess the impact of cardiovascular diseases and to guide therapeutic strategies. The Short Form 36 Health Survey and the RAND 36-Item Health Survey questionnaires are common tools for measuring health-related quality of life in patients with cardiovascular disease, but their reliability may vary according to the population studied. The aim of this study is to compare the reliability of the SF-36 and the RAND-36 in a population with cardiac pathology, addressing the question of which of these instruments offers a more consistent and useful measurement in this specific group. **Methods**: A cross-sectional observational study was carried out at the University Hospital of Burgos (Spain). A total of 413 patients with cardiovascular pathology referred to the Cardiac Rehabilitation Unit were included. Patients with incomplete data or who did not participate in the program were excluded. Internal consistency (Cronbach’s alpha), item–total correlation and reliability, and a half-and-half analysis were performed. **Results**: Both questionnaires showed similar and adequate reliability for patients with cardiovascular pathology. Internal consistency, as measured with Cronbach’s alpha, was above 0.80 for most dimensions, supporting its robustness. Significant inter-item and inter-dimension correlations were found in both scales, except in some specific cases in the dimension ‘Physical Functioning’. The half-and-half analysis confirmed the good reliability of both scales. **Conclusions**: Both the SF-36 and the RAND-36 are highly reliable tools for assessing health-related quality of life in patients with cardiovascular disease. The results may have significant implications for clinical practice, helping in the selection of health-related quality of life monitoring instruments and in the evaluation of the efficacy of therapeutic interventions.

## 1. Introduction

Cardiovascular disease (CVD) is one of the leading causes of morbidity and mortality worldwide [1], significantly affecting the quality of life of those who suffer from it. According to the World Health Organization (WHO), CVD is the leading cause of death, with an estimated 17.9 million deaths per year globally, accounting for approximately 32% of all recorded deaths [2]. Poor cardiovascular health impacts not only life expectancy, but also patients’ functional capacity, emotional well-being, and quality of life [1].

Health-related quality of life (HRQoL) is a term that aims to evaluate quality of life in relation to the effects of health, disease, and treatment; it is a multidimensional concept that includes physical, mental, and social well-being and has become a key indicator in the evaluation of the outcomes of medical interventions in patients with chronic diseases, such as cardiac pathologies [3]. Assessing HRQoL in patients with cardiac pathology is essential, as it allows for not only understanding the impact of the disease on patients’ daily lives but also guiding therapeutic strategies to improve their overall well-being.

The assessment of HRQoL in patients with CDV is crucial to understand the impact of these pathologies on the overall well-being of individuals. In this context, self-assessment questionnaires have become essential tools to measure how these conditions affect HRQoL. Two of the most widely used instruments in this area are the Short Form 36 Health Survey (SF-36) and the RAND 36-Item Health Survey (RAND-36), which, although very similar in design, may differ in reliability and validity in different populations.

Recent studies have highlighted the importance of factors such as adherence to treatment, social support, and comorbidity in the HRQoL of patients with heart disease [4,5]. Patient health status is also a predictor of other health outcomes, such as mortality, cardiovascular events, hospitalization, and healthcare costs; it is also useful for clinical decision-making, targeting healthcare resources, and enabling accurate patient monitoring [4]. Patient HRQoL is therefore important as a predictor of adverse events, as an indicator, and as a health outcome [4].

The SF-36 scale is one of the most widely used instruments worldwide to measure HRQoL, providing a comprehensive assessment of patients’ perception of their physical and mental health [6,7]. This multidimensional questionnaire addresses eight dimensions of health: physical function, physical role, body pain, general health, vitality, social function, emotional role, and mental health and also includes a single item that provides an indication of perceived change in health [8].

In the context of Cardiology, the SF-36 scale has proven to be a useful tool for assessing the impact of heart disease on patients’ HRQoL. Numerous studies have used it in patients with various heart diseases, including heart failure, ischemic heart disease, and valvular heart disease, among others [7,9,10]. These assessments are essential to better understand the functional and emotional limitations experienced by patients, as well as to guide therapeutic decisions and improve long-term prognosis.

It is a tool widely recognized for its robustness and validation in diverse populations and diseases that has demonstrated good psychometric properties, with high internal consistency and the ability to detect changes in perceived health over time [6,7,11,12]. The SF-36 was translated, adapted, and validated in Spanish [7,13,14,15].

On the other hand, the RAND-36 is an adaptation of the SF-36 developed by the RAND Corporation, which aims to provide a simplified and straightforward version of the original instrument [8,16]; it may currently be the most widely used HRQoL instrument worldwide [16]. Although the RAND-36 shares the dimensions of the SF-36 and includes the same item on perceived change in health [8], its major differences lie in the way the score is calculated which may influence its ability to accurately capture HRQoL in specific populations [16]. The distinct advantage of the RAND-36 lies in its availability and flexibility in the interpretation of its items, which makes it particularly useful in clinical research. The RAND-36 is available in the same languages as the SF-36 as the items have the same wording [16].

For both scales, it is also possible to calculate two summary scores, physical health and mental health, which are derived from the eight dimensions [8,16].

Thus, the choice between the SF-36 and the RAND-36 may depend on factors such as the reliability of each questionnaire.

Reliability is a crucial aspect in the evaluation of HRQoL questionnaires, as it ensures that measurements are consistent and reproducible. In the context of cardiac pathology, where symptoms and disease progression can vary considerably between individuals, it is especially important that measurement instruments are sensitive and reliable. Several studies have addressed the reliability of the SF-36 and RAND-36 in different settings, but direct comparison of these instruments in a cardiac pathology population remains an area of needed research.

This study aims to compare the reliability of the SF-36 and the RAND-36 in a population with cardiac pathology, addressing the question of which of these instruments offers a more consistent and useful measurement in this specific group.

Thus, the main objective of this research is to compare the reliability of the SF-36 and the RAND-36 in a population with cardiac pathology. Specifically, the aim is to evaluate the internal consistency of both questionnaires (Cronbach’s alpha); to analyze the item–total correlation for each of the dimensions and the correlation between dimensions; and to compare the reliability of the questionnaires by means of a half-and-half analysis.

The comparison of these two questionnaires will not only allow for an understanding of how each instrument performs in the context of heart disease and to assess its reliability in a specific population but will also facilitate the identification of possible areas for improvement in the HRQoL measurement instruments. The information obtained may have significant implications for clinical practice, including the selection of instruments for monitoring HRQoL in patients with heart disease and assessing the efficacy of therapeutic interventions.

## 2. Materials and Methods

### 2.1. Participants

The participants were those patients with some type of cardiac pathology who attended a first Cardiac Rehabilitation consultation with the Physical Medicine and Rehabilitation specialist (Cardiac Rehabilitation Unit), after being referred by the Cardiologist responsible for the Cardiac Rehabilitation Unit, to assess their inclusion in the physical training carried out on an outpatient basis within the Cardiac Rehabilitation Program.

The inclusion criteria were to have a diagnosis of a cardiovascular pathology, to come to the Cardiac Rehabilitation consultation referred by Cardiology, and to sign the informed consent form drawn up for this study. Patients unable to take part in the Physical Exercise Program due to any pathology and those who refused to participate in the program were excluded.

Data were collected from 510 patients, of whom 97 whose data were incomplete were discarded, leaving a total of 413 patients who were included in this study.

This study was approved by the Medicines Research Ethics Committee of the Burgos and Soria Health Area (Ref. CEIm 2569) on 22 June 2021. The recommendations set out in the Declaration of Helsinki of the World Medical Association were followed for this research.

### 2.2. Procedure

A cross-sectional observational study was carried out on the reliability of the SF-36 and RAND-36 scales applied to a sample of patients with CVD. No sample calculation was performed, and there is no control group for this research.

Once the inclusion criteria had been checked, the data on each patient were collected at the Rehabilitation Department of the University Hospital of Burgos at the time of the initial consultation with patients who are likely to be included in a Cardiac Rehabilitation Program. The researcher who collected the data is a physician specializing in physical medicine and rehabilitation; clinical data were taken from the patients’ medical records, and sociodemographic data and HRQoL assessment scales were collected at the consultation. Once the data were collected, they were entered into a database for further statistical analysis.

The variables collected were biological gender, place of residence, age, occupation, existence or not of risk factors (arterial hypertension, dyslipidemia, diabetes mellitus, active smoking, alcohol, other toxic substances, and physical inactivity), diagnosis of cardiac pathologies (ischemic heart disease, heart failure, valvular heart disease, arrhythmia, implanted device, chronic ischemic heart disease, ventricular dysfunction, and others), height (cm), weight (kg), muscle mass index (kg/m^2^), abdominal circumference (cm), blood pressure, and HRQoL (SF-36 and RAND-36 scales). Some of the variables are referred by the patient and others are objectified in the consultation room.

HRQoL is measured with the Spanish version of the SF-36 and the Spanish version of the RAND-36 scales. Both scales are composed of the same 36 items. The SF-36 was originally developed to be a generic instrument [17], whereas the RAND-36 is derived from the SF-36 but with differences in the coding of some items and in the handling of missing data [12,17]. In the case of the SF-36, each item must be recoded after which the raw scores for each of the 8 dimensions are calculated and, finally, these raw scores need to be transformed to a scale from 0 to 100. The calculation of the RAND-36 scores is significantly simpler; the scores for each item are recoded, and for the calculation of the dimensions, an average of the items answered in each dimension is made.

The treatment of missing data for the SF is carried out by filling in the data that the patient has not answered with the average of the scores of the same dimension. In the RAND-36, the missing data of the items are not filled in, as the average is used to calculate the score for each dimension. In both cases, it is pointed out that this imputation is possible as long as the missing data in the same dimension are not too many; therefore, this has been taken into account for this study.

Slight differences are also found in the naming of the dimensions; however, these differences only concern the name of some of the dimensions. Appendix A shows the equivalences between them, but it should be taken into account that there is literature in which the same terms are used for the RAND-36 dimensions as the original SF-36 ones [16,18,19]. Therefore, both tools are divided into 8 health dimensions that score between 0 and 100 plus an item related to reported health transition that compares the perceived health at the time of completing the survey with the situation a year before, but which is not included in any of the dimensions. Two summary scores can also be made, related to physical health and mental health that are derived from the previous dimensions [20,21].

### 2.3. Statistical Analysis

The statistical analysis was carried out with the IBM-SPSS V.25.0 program (IBM Corp., Armonk, NY, USA, EE. UU.

Firstly, a descriptive analysis of the variables was carried out. Quantitative variables are described with the mean and standard deviation and qualitative variables with the distribution of frequencies and percentages. This analysis aims to describe the study sample for a better understanding of this study.

Subsequently, a comparative analysis of the reliability of both scales was carried out. This involved assessing the internal consistency of both questionnaires, using Cronbach’s alpha coefficient to determine the homogeneity of responses within each dimension of the SF-36 and RAND-36 questionnaires; analyzing the item–total correlation for each of the dimensions and the correlation between the dimensions, in order to examine how each item relates to the total dimension and assessing the internal consistency of the dimensions; and comparing the reliability of the questionnaires by means of half-and-half analysis.

The scores of both tools used for the analyses were item-corrected scores and transformed scores for the dimensions on a case-by-case basis following the scoring norms of each of the scales.

## 3. Results

### 3.1. Descriptive Analysis

A total of 413 people with CVD who were treated at the Cardiac Rehabilitation Unit of the Rehabilitation Service of the University Hospital of Burgos participated in this study. Of these, 352 (85.2%) were male and 61 (14.8%) were female; 300 (72.6%) lived in the city of Burgos and 113 (27.4%) in the province of Burgos. The mean age of the participants was 60.27 years (SD = 10.86) with a maximum of 15 and a minimum of 84 years.

Table 1 shows the descriptive statistics for the rest of the qualitative variables related to risk factors and diagnosis.

Table 2 shows the descriptive statistics for the rest of the quantitative variables.

### 3.2. Results for Internal Consistency: Cronbach’s Alpha

Table 3 shows a comparison of the Cronbach’s alpha value for each of the dimensions of the SF-36 and RAND-36 tools and for the complete tools. First, for each dimension, the items that make up each dimension were introduced into the analysis. Secondly, for the Cronbach’s alpha of the complete tool, the 36 items were included, on the one hand, and the eight dimensions of each scale, on the other hand.

Moreover, in all the analyses, the Cronbach’s Alpha if an element is removed is similar to that obtained, so that it would not be appropriate to remove any of them in any of the analyses carried out.

### 3.3. Correlations between Items and Items—Total Score

For each of the eight dimensions in both scales, the item and item–total correlations of the dimension are always significant, except within the Physical Functioning dimension, in the correlation between items 3a and 3i and between items 3a and 3j, where there is no significant correlation in any of the scales. These correlation matrices are shown in Supplementary Material S1.

In addition, the eight dimensions also correlate significantly with each other for each of the scales, as shown in Table 4 and Table 5.

### 3.4. Half-and-Half Method

Finally, reliability statistics using the half-and-half method were examined.

The comparative reliability of the SF-36 and RAND-36 tools was also examined using the half-and-half method by introducing the 36 items into the analysis, as shown in Table 6, and by introducing the eight dimensions into the analysis, as shown in Table 7. The distribution of the items in the two halves was realized automatically with the statistical program, as the items are of similar difficulty. Both cases indicated good reliability.

## 4. Discussion

In the present study, the reliability of the SF-36 and RAND-36 scales were compared in a sample of patients with CVD, using various statistical methods. The results show that both scales, SF-36 and RAND-36, have a similar and adequate reliability for the sample of patients with cardiac pathology. The internal consistency of the complete scales and their dimensions is good as shown by the Cronbach’s alpha values in all cases. Furthermore, there is a correlation between the items of each dimension in all cases except within the Physical Functioning dimension, in the correlation between items 3a and 3i and between items 3a and 3j, where there is no significant correlation in any of the scales; however, the item–total correlation is significant in all dimensions for both scales. The correlation between the scores of all dimensions is also significant. The reliability assessed via the half-and-half method also yields results that confirm the good reliability of both scales. No differences were detected between the scales in any of these values.

The demographic profile of the sample in this study, comprising 85.2% men and a mean age of 60.27 years, is consistent with the previous literature on CVD. Several studies have documented that CVD, especially coronary heart disease, tends to be more prevalent in men than in women [22,23,24,25,26]. Women are twice as likely to have good cardiovascular health as men [27].

However, this demographic profile also highlights a common limitation in cardiovascular research: the under-representation of women, which could influence the generalizability of the results [25,26,28]. It is important that future studies seek to balance gender representation to better understand how these scales function in women with CVD, who often present with different clinical manifestations and responses to treatments. [26,28,29].

Internal consistency, assessed with Cronbach’s alpha, showed values above 0.80 for the full scale including all 36 items, for the full scale including all eight dimensions and for most dimensions in both scales. This finding is consistent with previous research reporting that both the SF-36 and the RAND-36 are robust instruments for measuring HRQoL in patients with chronic diseases, including CVD [6,7,8,11,12,15,16,17,30,31,32,33]. The general health dimension showed a Cronbach’s alpha of 0.756 in the SF-36 and 0.746 in the RAND-36, and the social functioning dimension exhibited a value of 0.666 in the SF-36 and 0.773 in the RAND-36; which, although lower scores, are acceptable values.

The RAND-36 can be considered an alternative version of the MOS SF-36; thus, both instruments must meet the same psychometric standards as established by Ware and Sherbourne [6] who considered that the most important criteria for the MOS SF-36 are that the reliabilities must be acceptable and that the correlation between items for an item should be higher with its own subscale than with the other subscales of the instrument [17]. In this research, regarding item and item–total correlations, the results show that both the SF-36 and the RAND-36 have a coherent internal structure, with item–total correlations exceeding the recommended threshold of 0.30 [34]. This implies that each item contributes adequately to the overall measure of the dimension to which it belongs, which supports the internal validity of both scales. These correlations reinforce the idea that items within each dimension are measuring similar constructs, which is essential for the clinical interpretation of the scores obtained [6,11,12,15,32,33].

Reliability analysis using the half-and-half method also showed positive results for both scales, with coefficients indicating high measurement stability. This is particularly relevant in clinical settings, where it is crucial that assessment tools are able to provide consistent results over time and across different population subgroups [35]. Consistency in measurement supports the use of these instruments in daily clinical practice to assess HRQoL in patients with CVD.

Despite the similarities in reliability between the two scales, it is important to highlight some theoretical and methodological differences that could influence the selection of one over the other in future research. The SF-36 was originally developed as a generic instrument, applicable to a wide range of health conditions and populations, while the RAND-36 is derived from the SF-36 but with differences in the coding of certain items and the handling of missing data [16]. However, the results of this study suggest that, in terms of reliability, both scales are interchangeable in assessing HRQoL in patients with CVD.

This study presents several limitations that should be considered when interpreting the results. First, the sample consisted exclusively of patients with CVD, which limits the generalization of the findings to other populations with different health conditions. Previous studies have indicated that the psychometric properties of the SF-36 and RAND-36 scales may vary depending on the population studied, particularly when dealing with different chronic diseases or healthy individuals [36,37].

Second, although a comprehensive comparative analysis of reliability was conducted, this study did not include an assessment of the convergent or discriminant validity of the scales. Validity is a critical component to ensure that the scales effectively measure what they are intended to measure [38], and its absence in this analysis limits the full interpretation of the utility of each tool. Sensitivity to change, an essential property for determining the ability of the scales to detect variations in health status over time, was also not explored, which could be especially relevant in monitoring patients with chronic diseases.

Finally, the cross-sectional design of this study prevents establishing causal relationships between the observed psychometric properties and the health condition of the patients. The cross-sectional nature limits the ability to assess the stability of psychometric properties over time. Longitudinal studies would be necessary to confirm the stability of these properties at different stages of CVD and in other chronic conditions [39].

This study presents several strengths that reinforce the validity of its findings. First, one of the main strengths is the use of a rigorous methodological approach to evaluate the reliability of the SF-36 and RAND-36 scales in a specific sample of patients with CVD. The use of multiple statistical methods, such as Cronbach’s alpha, item–total correlation, and hand-and-half reliability analysis, provides a comprehensive and robust view of the internal consistency of these scales. The diversity of analyses used allows for a more thorough assessment of reliability, which increases confidence in the results obtained [40].

Sample size is a crucial aspect that can influence both the strengths and limitations of a study. In this case, the use of a sample of 413 patients with CVD may represent a strength of this study. An adequate sample size allows for more precise estimates of psychometric properties, such as reliability, and ensures greater statistical power to detect significant differences and relationships in the analyses [41]. Additionally, with a sample of this size, the external validity of the results is improved, making the conclusions more generalizable to the broader population with CVD [42]. However, the majority of patients had ischemic heart disease (95.4%). Therefore, the analysis might be valid only for these patients and might not be generalizable to other patients with heart failure, valvular heart disease, or ischemic heart disease.

This research allows for a detailed and relevant assessment of the reliability of the scales in a context of great clinical importance, making the results directly applicable to practice in the cardiovascular field. The specificity of the sample provides a solid foundation for the clinical interpretation of the psychometric properties of the scales in patients with CVD, which is crucial for the personalization of care and follow-up of these patients [43]. Nonetheless, it is important to note that the analysis was conducted before patients with cardiac conditions began the rehabilitation process, which means that the findings are specific to this population of patients who have been admitted to the cardiac rehabilitation service due to their underlying conditions. While this allows for an accurate assessment of health-related quality of life (HRQoL) in this context, it also limits the generalizability of the results to other patient populations with different cardiac conditions. Future research would benefit from exploring HRQoL in groups of patients with single causes of rehabilitation, which would provide a better understanding of variations in quality of life among different clinical entities.

Additionally, this study contributes to the field by comparing two widely used scales, the SF-36 and the RAND-36, facilitating informed decision-making about which tool is more suitable in different research and clinical practice contexts. The direct comparison between these two scales under the same conditions and within the same population is a valuable contribution that allows researchers and clinicians to select the most appropriate scale according to their specific needs.

From the findings of this study, several lines of research can be identified that could further enrich the knowledge about the assessment of HRQoL in patients with CVD. These include studying the sensitivity to change to detect variations in patients’ quality of life as their disease progresses or as they respond to therapeutic interventions; validation in other cultural and demographic contexts, such as different ethnic groups, socioeconomic levels, or patients from various geographic regions; and research on the predictive validity of long-term clinical outcomes, such as morbidity, mortality, or rehospitalization in patients with CVD. This could make these scales more powerful tools for clinical management and treatment planning. Future research should also aim to explore the health-related quality of life (HRQoL) in patients with various cardiovascular conditions, including heart failure and valvular heart disease, to determine if the findings observed in patients with ischemic heart disease are applicable to these other patient groups. This could involve a comparative analysis across different cardiovascular diseases to better understand the nuances and specificities of HRQoL in these populations.

## 5. Conclusions

The present study has demonstrated that both the SF-36 and RAND-36 scales exhibit high reliability in assessing HRQoL in patients with CVD. Through various statistical methods, including Cronbach’s alpha, item–total correlations, and half-and-half reliability analysis, it has been confirmed that both scales are consistent and suitable tools for use in this population. These findings suggest that the two scales can be used interchangeably in clinical and research contexts for evaluating HRQoL in patients with CVD.

Given that both scales have shown consistency and reliability in this population, the choice between them may depend on other factors such as availability, the clinical staff’s familiarity with the instrument, or specific research considerations. However, even though the reliability of both scales is comparable, future research could explore other aspects, such as sensitivity to change and the predictive capacity of clinical outcomes, which could help determine the most appropriate scale for different clinical or research objectives.

In summary, both the SF-36 and RAND-36 represent viable and robust options for measuring HRQoL in patients with CVD, supporting their usefulness in clinical practice and epidemiological studies.

## Figures and Tables

**Table 1:** Descriptive statistics of the qualitative variables.

		Frequency	%
Occupation/job	White collar	44	10.7
	Blue collar	197	47.7
	Retired	140	33.9
	Unemployed	6	1.5
	Permanent work disability	5	1.2
	House holder	15	3.6
	No data	6	1.5
AHT	YES	208	50.4
	NO	205	49.6
Dyslipidaemia	YES	226	54.7
	NO	187	45.3
DM	YES	103	24.9
	NO	308	74.6
	No data	2	0.5
Active smoking	YES	75	18.2
	NO	338	81.8
Alcohol	YES	1	0.2
	NO	411	99.5
	No data	1	0.2
Other intoxicants	YES	8	1.9
	NO	405	98.1
Physical inactivity	YES	108	26.2
	NO	294	71.2
	No data	11	2.7
Ischemic heart disease	YES	394	95.4
	NO	19	4.6
Heart failure	YES	12	2.9
	NO	401	97.1
Interventional valve disease	YES	20	4.8
	NO	393	95.2
Arrhythmia	YES	51	12.3
	NO	362	87.7
Implanted device (pacemaker/implantable cardioverter-defibrillator)	YES	32	7.7
	NO	381	92.3
Chronic ischemic heart disease	YES	59	14.3
	NO	354	85.7
Ventricular dysfunction	YES	76	18.4
	NO	336	81.4
	No data	1	0.2
Other cardiac pathologies	YES	51	12.3
	NO	362	87.7

AHT: arterial hypertension; DM: diabetes mellitus.

**Table 2:** Descriptive statistics of quantitative variables.

	N	Minimum	Maximum	Mean	SD
Height (cm)	413	147	193	168.22	7.986
Weight Kg)	413	8	135	78.31	14.677
BMI (Kg/m^2^)	413	18	44	27.77	4.281
Abdominal circumference (cm)	402	67	186	100.49	12.201
Systolic blood pressure	411	80	180	131,55	15.638
Diastolic blood pressure	411	49	100	77.90	7.627

SD: standard deviation; BMI: body mass index.

**Table 3:** Cronbach’s alphas.

		Cronbach’s Alpha	
SF-36 Terminology	RAND-36 Terminology	SF-36	RAND-36
Physical Functioning (PF) items	Physical Functioning items	0.875	0.877
Role Physical (RP) items	Role limitations due to physical health	0.870	0.872
Bodily Pain (BP) items	Pain	0.859	0.816
General Health (GH) items	General health	0.756	0.746
Vitality (VT) items	Energy/Fatigue	0.816	0.816
Social Functioning (SF) items	Social functioning	0.666	0.773
Role Emotional (RE) items	Role limitations due to emotional problems	0.916	0.916
Mental Health (MH) items	Emotional well-being	0.874	0.874
Complete tool (36 items)	Complete tool (36 items)	0.926	0.937
Complete tool (8 dimensions)	Complete tool (8 dimensions)	0.838	0.846

**Table 4:** Correlations between the SF-36 dimensions.

		SF_PF	SF_RP	SF_BP	SF_GH	SF_VT	SF_SF	SF_RE	SF_MH
SF_PF	Pearson correlation	1	0.409 **	0.492 **	0.507 **	0.556 **	0.387 **	0.309 **	0.362 **
	Sig. (bilateral)		<0.001	<0.001	<0.001	<0.001	<0.001	<0.001	<0.001
	N	406	395	401	396	394	393	395	394
SF_RP	Pearson correlation	0.409 **	1	0.428 **	0.380 **	0.494 **	0.400 **	0.395 **	0.361 **
	Sig. (bilateral)	<0.001		<0.001	<0.001	<0.001	<0.001	<0.001	<0.001
	N	395	401	396	390	388	387	395	388
SF_BP	Pearson correlation	0.492 **	0.428 **	1	0.409 **	0.484 **	0.419 **	0.387 **	0.401 **
	Sig. (bilateral)	<0.001	<0.001		<0.001	<0.001	<0.001	<0.001	<0.001
	N	401	396	407	399	397	396	400	396
SF_GH	Pearson correlation	0.507 **	0.380 **	0.409 **	1	0.572 **	0.415 **	0.382 **	0.477 **
	Sig. (bilateral)	<0.001	<0.001	<0.001		<0.001	<0.001	<0.001	<0.001
	N	396	390	399	402	398	395	394	395
SF_VT	Pearson correlation	0.556 **	0.494 **	0.484 **	0.572 **	1	0.565 **	0.448 **	0.664 **
	Sig. (bilateral)	<0.001	<0.001	<0.001	<0.001		<0.001	<0.001	<0.001
	N	394	388	397	398	400	394	392	395
SF_SF	Pearson correlation	0.387 **	0.400 **	0.419 **	0.415 **	0.565 **	1	0.574 **	0.640 **
	Sig. (bilateral)	<0.001	<0.001	<0.001	<0.001	<0.001		<0.001	<0.001
	N	393	387	396	395	394	398	391	393
SF_RE	Pearson correlation	0.309 **	0.395 **	0.387 **	0.382 **	0.448 **	0.574 **	1	0.662 **
	Sig. (bilateral)	<0.001	<0.001	<0.001	<0.001	<0.001	<0.001		<0.001
	N	395	395	400	394	392	391	401	390
SF_MH	Pearson correlation	0.362 **	0.361 **	0.401 **	0.477 **	0.664 **	0.640 **	0.662 **	1
	Sig. (bilateral)	<0.001	<0.001	<0.001	<0.001	<0.001	<0.001	<0.001	
	N	394	388	396	395	395	393	390	399

**. Correlation is significant at the 0.01 level (bilateral).

**Table 5:** Correlations between RAND-36 dimensions.

		RAND_PF	RAND_RP	RAND_BP	RAND_GH	RAND_VT	RAND_SF	RAND_RE	RAND_MH
**RAND_PF**	Pearson correlation	1	0.422 **	0.522 **	0.518 **	0.552 **	0.450 **	0.313 **	0.361 **
	Sig. (bilateral)		<0.001	<0.001	<0.001	<0.001	<0.001	<0.001	<0.001
	N	387	371	381	377	378	375	377	378
**RAND_RP**	Pearson correlation	0.422 **	1	0.442 **	0.384 **	0.499 **	0.404 **	0.402 **	0.365 **
	Sig. (bilateral)	<0.001		<0.001	<0.001	<0.001	<0.001	<0.001	<0.001
	N	371	395	390	383	382	381	389	383
**RAND_BP**	Pearson correlation	0.522 **	0.442 **	1	0.422 **	0.493 **	0.473 **	0.390 **	0.413 **
	Sig. (bilateral)	<0.001	<0.001		<0.001	<0.001	<0.001	<0.001	<0.001
	N	381	390	406	397	396	395	400	395
**RAND_GH**	Pearson correlation	0.518 **	0.384 **	0.422 **	1	0.566 **	0.444 **	0.383 **	0.476 **
	Sig. (bilateral)	<0.001	<0.001	<0.001		<0.001	<0.001	<0.001	<0.001
	N	377	383	397	401	397	394	393	394
**RAND_VT**	Pearson correlation	0.552 **	0.499 **	0.493 **	0.566 **	1	0.601 **	0.448 **	0.664 **
	Sig. (bilateral)	<0.001	<0.001	<0.001	<0.001		<0.001	<0.001	<0.001
	N	378	382	396	397	400	394	392	395
**RAND_SF**	Pearson correlation	0.450 **	0.404 **	0.473 **	0.444 **	0.601 **	1	0.551 **	0.668 **
	Sig. (bilateral)	<0.001	<0.001	<0.001	<0.001	<0.001		<0.001	<0.001
	N	375	381	395	394	394	398	391	393
**RAND_RE**	Pearson correlation	0.313 **	0.402 **	0.390 **	0.383 **	0.448 **	0.551 **	1	0.662 **
	Sig. (bilateral)	<0.001	<0.001	<0.001	<0.001	<0.001	<0.001		<0.001
	N	377	389	400	393	392	391	401	390
**RAND_MH**	Pearson correlation	0.361 **	0.365 **	0.413 **	0.476 **	0.664 **	0.668 **	0.662 **	1
	Sig. (bilateral)	<0.001	<0.001	<0.001	<0.001	<0.001	<0.001	<0.001	
	N	378	383	395	394	395	393	390	399

**. Correlation is significant at the 0.01 level (bilateral).

**Table 6:** Reliability statistics of half-and-half method with 36 items.

			SF-36	RAND-36
Cronbach´s Alpha	Part 1	Value	0.875	0.894
		N of elements	18 ^a^	18 ^a^
	Part 2	Value	0.890	0.913
		N of elements	18 ^b^	18 ^b^
	N total of elements		36	36
Correlation between forms			0.679	0.666
Spearman–Brown Coefficient	Equal Length		0.809	0.799
	Unequal Length		0.809	0.799
Guttman two halves coefficient			0.776	0.799

^a^. The elements are SF3a, SF3b, SF3c, SF3d, SF3e, SF3f, SF3g, SF3h, SF3i, SF3j, Sf4a, SF4b, SF4c, SF4d, SF7, SF8, SF1, and SF11a for SF-36; and RAND3a, RAND3b, RAND3c, RAND3d, RAND3e, RAND3f, RAND3g, RAND3h, RAND3i, RAND3j, RAND4a, RAND4b, RAND4c, RAND4d, RAND7, RAND8, RAND1, and RAND11a for RAND-36. ^b^. The elements are SF11b, SF11c, SF11d, SF9a, SF9e, SF9g, SF9i, SF6, SF10, SF5a, SF5b, SF5c, SF9b, SF9c, SF9d, SF9f, SF9h, and SF2 for SF-36; and RAND11b, RAND11c, RAND11d, RAND9a, RAND9e, RAND9g, RAND9i, RAND6, RAND10, RAND5a, RAND5b, RAND5c, RAND9b, RAND9c, RAND9d, RAND9f, RAND9h, and RAND2 for RAND-36.

**Table 7:** Reliability statistics of half-and-half method with 8 dimensions.

			SF-36	RAND-36
Cronbach´s Alpha	Part 1	Value	0.722	0.729
		N of elements	4 ^a^	4 ^a^
	Part 2	Value	0.773	0.780
		N of elements	4 ^b^	4 ^b^
	N total of elements		8	8
Correlation between forms			0.646	0.666
Spearman–Brown Coefficient	Equal Length		0.785	0.799
	Unequal Length		0.785	0.799
Guttman two halves coefficient			0.779	0.799

^a^. The elements are SF_PF, SF_RP, SF_BP, and SF_GH for SF-36; and RAND_PF, RAND_RP, RAND_BP, and RAND_GH for RAND-36. ^b^. The elements are SF_VT, SF_SF, SF_RE, and SF_MH for SF-36; and RAND_VT, RAND_SF, RAND_RE, and RAND_MH for RAND-36.

## Data Availability

The datasets presented in this article are not readily available because the data are part of an ongoing study. Requests to access the datasets should be directed to mspelaez@ubu.es.

## Supplementary Materials

The following supporting information can be downloaded at: https://www.mdpi.com/article/10.3390/jcm13206106/s1, Supplementary Material S1: Item-item and item-total correlation matrices for each of the eight dimensions in both scales.

## Appendix A. Relationship between SF-36 and RAND-36 Terminology

**SF-36 Terminology**	**RAND-36 Terminology**	**Items**
Physical Functioning (PF)	Physical Functioning	3a, 3b, 3c, 3d, 3e, 3f, 3g, 3h, 3i, 3j
Role Physical (RP)	Role limitations due to physical health	4a, 4b, 4c, 4d
Bodily Pain (BP)	Pain	7, 8
General Health (GH)	General health	1, 11a, 11b, 11c, 11d
Vitality (VT)	Energy/Fatigue	9a, 9e, 9g, 9i
Social Functioning (SF)	Social functioning	6, 10
Role Emotional (RE)	Role limitations due to emotional problems	5a, 5b, 5c
Mental Health (MH)	Emotional well-being	9b, 9c, 9d, 9f, 9h
Reported health transition item	Reported health transition item	2
