# Supplementary material for: Comparison of SF-36 and RAND-36 in Cardiovascular Diseases: A Reliability Study
Source: J Clin Med. 2024 Oct 13;13(20):6106. doi: 10.3390/jcm13206106 (PMC11508691; doi:10.3390/jcm13206106)
Supplement: Supplementary file 1 [file jcm-13-06106-s001.zip › jcm-3210685-supplementary.pdf]

SUPPLEMENTARY MATERIAL S1

Item-item and item-total correlation matrices for each of the eight dimensions in both scales.

Table. Item-item and item-total correlation matrices for SF-36 “Physical Functioning” dimension.

		SF3a	SF3b	SF3c	SF3d	SF3e	SF3f	SF3g	SF3h	SF3i	SF3j	SF_PF
**SF3a**	Pearson correlation	1	.491**	.393**	.482**	.287**	.301**	.288**	.242**	.095	.049	.,558**
	Sig. (bilateral)		<.001	<.001	<.001	<.001	<.001	<.001	<.001	**.054**	**.319**	<.001
	N	411	410	409	410	411	411	411	409	409	411	406
**SF3b**	Pearson correlation	.491**	1	.637**	.563**	.509**	.503**	.522**	.416**	.269**	.198**	.766**
	Sig. (bilateral)	<.001		<.001	<.001	<.001	<.001	<.001	<.001	<.001	<.001	<.001
	N	410	411	409	410	411	411	411	409	409	411	406
**SF3c**	Pearson correlation	.393**	.637**	1	.511**	.515**	.384**	.447**	.433**	.332**	.270**	.722**
	Sig. (bilateral)	<.001	<.001		<.001	<.001	<.001	<.001	<.001	<.001	<.001	<.001
	N	409	409	411	409	411	410	411	408	408	411	406
**SF3d**	Pearson correlation	.482**	.563**	.511**	1	.579**	.494**	.491**	.407**	.236**	.134**	.747**
	Sig. (bilateral)	<.001	<.001	<.001		<.001	<.001	<.001	<.001	<.001	<.001	<.001
	N	410	410	409	411	411	411	411	410	410	411	406
**SF3e**	Pearson correlation	.287**	.509**	.515**	.579**	1	.529**	.618**	.546**	.532**	.361**	.781**
	Sig. (bilateral)	<.001	<.001	<.001	<.001		<.001	<.001	<.001	<.001	<.001	<.001
	N	411	411	411	411	413	412	413	410	410	413	406
**SF3f**	Pearson correlation	.301**	.503**	.384**	.494**	.529**	1	.506**	.468**	.361**	.341**	.723**
	Sig. (bilateral)	<.001	<.001	<.001	<.001	<.001		<.001	<.001	<.001	<.001	<.001
	N	411	411	410	411	412	412	412	410	410	412	406
**SF3g**	Pearson correlation	.288**	.522**	.447**	.491**	.618**	.506**	1	.692**	.548**	.330**	.780**
	Sig. (bilateral)	<.001	<.001	<.001	<.001	<.001	<.001		<.001	<.001	<.001	<.001

	N	411	411	411	411	413	412	.413	410	410	413	406
**SF3h**	Pearson correlation	.242**	.416**	.433**	.407**	.546**	.468**	.692**	1	.664**	.380**	.727**
	Sig. (bilateral)	<.001	<.001	<.001	<.001	<.001	<.001	<.001		<.001	<.001	<.001
	N	409	409	408	410	410	410	410	410	410	410	406
**SF3i**	Pearson correlation	.095	.269**	.332**	.236**	.532**	.361**	.548**	.664**	1	.562**	.599**
	Sig. (bilateral)	**,054**	<.001	<.001	<.001	<.001	<.001	<.001	<.001		<.001	<.001
	N	409	409	408	410	410	410	410	410	410	410	406
**SF3j**	Pearson correlation	.049	.198**	.270**	.134**	.361**	.341**	.330**	.380**	.562**	1	.449**
	Sig. (bilateral)	**,319**	<.001	<.001	,007	<.001	<.001	<.001	<.001	<.001		<.001
	N	411	411	411	411	413	412	413	410	410	413	406
**SF_PF**	Pearson correlation	.558**	.766**	.722**	.747**	.781**	.723**	.780**	.727**	.599**	.449**	1
	Sig. (bilateral)	<.001	<.001	<.001	<.001	<.001	<.001	<.001	<.001	<.001	<.001	
	N	406	406	406	406	406	406	406	406	406	406	406

**. The correlation is significant at the 0.01 level (bilateral). Bold: not significant correlations.

PF: Physical functioning.

Table. Item-item and item-total correlation matrices for SF-36 “Role Physical” dimension.

		Sf4a	SF4b	SF4c	SF4d	SF_RP
**Sf4a**	Pearson correlation	1	.602**	.657**	.572**	.841**
	Sig. (bilateral)		<.001	<.001	<.001	<.001
	N	405	403	403	405	401
**SF4b**	Pearson correlation	.602**	1	.624**	.621**	.828**
	Sig. (bilateral)	<.001		<.001	<.001	<.001
	N	403	404	402	404	.401
**SF4c**	Pearson correlation	.657**	.624**	1	.690**	,879**
	Sig. (bilateral)	<.001	<.001		<.001	<.001
	N	403	402	405	404	401
**SF4d**	Pearson correlation	.572**	.621**	.690**	1	.847**
	Sig. (bilateral)	<.001	<.001	<.001		<.001
	N	405	404	404	406	401
**SF_RP**	Pearson correlation	.841**	.828**	.879**	.847**	1
	Sig. (bilateral)	<.001	<.001	<.001	<.001	
	N	401	401	401	401	401

**. The correlation is significant at the 0.01 level (bilateral).

RP: Role physical.

Table. Item-item and item-total correlation matrices for SF-36 “Role Physical” dimension.

		SF7	SF8	SF_BP
**SF7**	Pearson correlation	1	.753**	.935**
	Sig. (bilateral)		<.001	<.001
	N	408	407	407
**SF8**	Pearson correlation	.753**	1	.938**
	Sig. (bilateral)	<.001		<.001
	N	407	408	407
**SF_BP**	Pearson correlation	.935**	.938**	1
	Sig. (bilateral)	<.001	<.001	
	N	407	407	407

**. The correlation is significant at the 0.01 level (bilateral).

BP: Bodily pain.

Table. Item-item and item-total correlation matrices for SF-36 “General Health” dimension.

		SF1	SF11a	SF11b	SF11c	SF11d	SF_GH
**SF1**	Pearson correlation	1	.348**	.438**	.341**	.511**	.704**
	Sig. (bilateral)		<.001	<.001	<.001	<.001	<.001
	N	411	402	402	402	402	402
**SF11a**	Pearson correlation	.348**	1	.339**	.350**	.294**	.664**
	Sig. (bilateral)	<.001		<.001	<.001	<.001	<.001
	N	402	404	404	404	404	402
**SF11b**	Pearson correlation	.438**	.339**	1	.254**	.646**	.768**
	Sig. (bilateral)	<.001	<.001		<.001	<.001	<.001
	N	402	404	404	404	404	402
**SF11c**	Pearson correlation	.341**	.350**	.254**	1	.368**	.637**
	Sig. (bilateral)	<.001	<.001	<.001		<.001	<.001
	N	402	404	404	404	404	402
**SF11d**	Pearson correlation	.511**	.294**	.646**	.368**	1	.796**
	Sig. (bilateral)	<.001	<.001	<.001	<.001		<.001
	N	402	404	404	404	404	402
**SF_GH**	Pearson correlation	.704**	.664**	.768**	.637**	.796**	1
	Sig. (bilateral)	<.001	<.001	<.001	<.001	<.001	
	N	402	402	402	402	402	402

**. The correlation is significant at the 0.01 level (bilateral).

GH: General health.

Table. Item-item and item-total correlation matrices for SF-36 “Vitality” dimension.

		SF9a	SF9e	SF9g	SF9i	SF_VT
**SF9a**	Pearson correlation	1	.706**	.402**	.445**	.812**
	Sig. (bilateral)		<.001	<.001	<.001	<.001
	N	403	401	402	402	400
**SF9e**	Pearson correlation	.706**	1	.425**	.506**	.837**
	Sig. (bilateral)	<.001		<.001	<.001	<.001
	N	401	404	403	403	400
**SF9g**	Pearson correlation	.402**	.425**	1	.693**	.765**
	Sig. (bilateral)	<.001	<.001		<.001	<.001
	N	402	403	405	405	400
**SF9i**	Pearson correlation	.445**	.506**	.693**	1	.801**
	Sig. (bilateral)	<.001	<.001	<.001		<.001
	N	402	403	405	405	400
**SF_VT**	Pearson correlation	.812**	.837**	.765**	.801**	1
	Sig. (bilateral)	<.001	<.001	<.001	<.001	
	N	400	400	400	400	400

**. The correlation is significant at the 0.01 level (bilateral).

VT: Vitality.

Table. Item-item and item-total correlation matrices for SF-36 “Social Functioning” dimension

		SF6	SF10	SF_SF
**SF6**	Pearson correlation	1	.515**	.902**
	Sig. (bilateral)		<.001	<.001
	N	407	398	398
**SF10**	Pearson correlation	.515**	1	.834**
	Sig. (bilateral)	<.001		<.001
	N	398	400	398
**SF_SF**	Pearson correlation	.902**	.834**	1
	Sig. (bilateral)	<.001	<.001	
	N	398	398	398

**. The correlation is significant at the 0.01 level (bilateral).

SF: Social functioning.

Table. Item-item and item-total correlation matrices for SF-36 “Role emotional” dimension

		**SF5a**	**SF5b**	**SF5c**	**SF_RE**
**SF5a**	Pearson correlation	1	.836**	.724**	.922**
	Sig. (bilateral)		<.001	<.001	<.001
	N	403	402	401	401
**SF5b**	Pearson correlation	.836**	1	.791**	.947**
	Sig. (bilateral)	<.001		<.001	<.001
	N	402	403	401	401
**SF5c**	Pearson correlation	.724**	.791**	1	.906**
	Sig. (bilateral)	<.001	<.001		<.001
	N	401	401	401	401
**SF_RE**	Pearson correlation	.922**	.947**	.906**	1
	Sig. (bilateral)	<.001	<.001	<.001	
	N	401	401	401	401

**. The correlation is significant at the 0.01 level (bilateral).

RE: Role emotional.

Table. Item-item and item-total correlation matrices for SF-36 “Mental Health” dimension

		SF9b	SF9c	SF9	SF9f	SF9h	SF_MH
**SF9b**	Pearson correlation	1	.590**	.618**	.525**	.405**	.769**
	Sig. (bilateral)		<.001	<.001	<.001	<.001	<.001
	N	405	403	405	402	403	399
**SF9c**	Pearson correlation	.590**	1	.580**	.754**	.559**	.856**
	Sig. (bilateral)	<.001		<.001	<.001	<.001	<.001
	N	403	403	403	400	402	399
**SF9d**	Pearson correlation	.618**	.580**	1	.541**	.610**	.823**
	Sig. (bilateral)	<.001	<.001		<.001	<.001	<.001
	N	405	403	406	402	404	399
**SF9f**	Pearson correlation	.525**	.754**	.541**	1	.595**	.839**
	Sig. (bilateral)	<.001	<.001	<.001		<.001	<.001
	N	402	400	402	402	400	399
**SF9h**	Pearson correlation	.405**	.559**	.610**	.595**	1	.791**
	Sig. (bilateral)	<.001	<.001	<.001	<.001		<.001
	N	403	402	404	400	404	399
**SF_MH**	Pearson correlation	.769**	.856**	.823**	.839**	.791**	1
	Sig. (bilateral)	<.001	<.001	<.001	<.001	<.001	
	N	399	399	399	399	399	399

**. The correlation is significant at the 0.01 level (bilateral).

MH: Mental health.

Table. Item-item and item-total correlation matrices for RAND-36 “Physical functioning” dimension.

		RAND3a	RAND3b	RAND3c	RAND3d	RAND3e	RAND3f	RAND3	RAND3h	RAND3i	RAND3	RAND_PF
**RAND3a**	Pearson correlation	1	.500**	.408**	.481**	.283**	.302**	.278**	.236**	.088	.041	.557**
	Sig. (bilateral)		<.001	<.001	<.001	<.001	<.001	<.001	<.001	**.078**	**.405**	<.001
	N	407	403	400	402	403	406	403	404	404	406	387
**RAND3b**	Pearson correlation	.500**	1	.644**	.563**	.511**	.504**	.521**	.415**	.269**	.190**	.780**
	Sig. (bilateral)	<.001		<.001	<.001	<.001	<.001	<.001	<.001	<.001	<.001	<.001
	N	403	408	401	403	403	407	404	405	405	407	387
**RAND3c**	Pearson correlation	.408**	.644**	1	.510**	.512**	.380**	.442**	.427**	.322**	.269**	.727**
	Sig. (bilateral)	<.001	<.001		<.001	<.001	<.001	<.001	<.001	<.001	<.001	<.001
	N	400	401	406	401	404	404	403	402	402	405	387
**RAND3d**	Pearson correlation	.481**	.563**	.510**	1	.586**	.499**	.492**	.414**	.239**	.134**	.758**
	Sig. (bilateral)	<.001	<.001	<.001		<.001	<.001	<.001	<.001	<.001	<.001	<.001
	N	402	403	401	407	403	406	405	405	405	407	387
**RAND3e**	Pearson correlation	.283**	.511**	.512**	.586**	1	.534**	.622**	.545**	.535**	.365**	.787**
	Sig. (bilateral)	<.001	<.001	<.001	<.001		<.001	<.001	<.001	<.001	<.001	<.001
	N	403	403	404	403	408	406	405	404	404	407	387
**RAND3f**	Pearson correlation	.302**	.504**	.380**	.499**	.534**	1	.502**	.467**	.360**	.337**	.723**
	Sig. (bilateral)	<.001	<.001	<.001	<.001	<.001		<.001	<.001	<.001	<.001	<.001
	N	406	407	404	406	406	411	407	408	408	410	387
**RAND3g**	Pearson correlation	.278**	.521**	.442**	.492**	.622**	.502**	1	.692**	.549**	.323**	.782**
	Sig. (bilateral)	<.001	<.001	<.001	<.001	<.001	<.001		<.001	<.001	<.001	<.001
	N	403	404	403	405	405	407	409	406	406	409	387
**RAND3h**	Pearson correlation	.236**	.415**	.427**	.414**	.545**	.467**	.692**	1	.662**	.364**	.721**
	Sig. (bilateral)	<.001	<.001	<.001	<.001	<.001	<.001	<.001		<.001	<.001	<.001
	N	404	405	402	405	404	408	406	409	409	408	387
**RAND3i**	Pearson correlation	.088	.269**	.322**	.239**	.535**	.360**	.549**	.662**	1	.550**	.596**

	Sig. (bilateral)	**.078**	<.001	<.001	<.001	<.001	<.001	<.001	<.001		<.001	<.001
	N	404	405	402	405	404	408	406	409	409	408	387
**RAND3j**	Pearson correlation	.041	.190**	.269**	.134**	.365**	.337**	.323**	.364**	.550**	1	.450**
	Sig. (bilateral)	**.405**	<.001	<.001	.007	<.001	<.001	<.001	<.001	<.001		<.001
	N	406	407	405	407	407	410	409	408	408	412	387
**RAND_PF**	Pearson correlation	.557**	.780**	.727**	.758**	.787**	.723**	.782**	.721**	.596**	.450**	1
	Sig. (bilateral)	<.001	<.001	<.001	<.001	<.001	<.001	<.001	<.001	<.001	<.001	
	N	387	387	387	387	387	387	387	387	387	387	387

**. The correlation is significant at the 0.01 level (bilateral). Bold: not significant correlations.

PF: Physical functioning.

Table. Item-item and item-total correlation matrices for RAND-36 “Role limitations due to physical heath” dimension.

		RAND4a	RAND4b	RAND4c	RAND4d	RAND_RP
**RAND4a**	Pearson correlation	1	.602**	.662**	.579**	.843**
	Sig. (bilateral)		<.001	<.001	<.001	<.001
	N	402	397	400	402	395
**RAND4b**	Pearson correlation	.602**	1	.628**	.628**	.831**
	Sig. (bilateral)	<.001		<.001	<.001	<.001
	N	397	400	398	400	395
**RAND4c**	Pearson correlation	.662**	.628**	1	.689**	.880**
	Sig. (bilateral)	<.001	<.001		<.001	<.001
	N	400	398	404	403	395
**RAND4d**	Pearson correlation	.579**	.628**	.689**	1	.848**
	Sig. (bilateral)	<.001	<.001	<.001		<.001
	N	402	400	403	405	395
**RAND_RP**	Pearson correlation	.843**	.831**	.880**	.848**	1
	Sig. (bilateral)	<.001	<.001	<.001	<.001	
	N	395	395	395	395	395

**. The correlation is significant at the 0.01 level (bilateral).

RP: Role limitations due to physical heath.

Table. Item-item and item-total correlation matrices for RAND-36 "Pain" dimension

		RAND7	RAND8	RAND_BP
**RAND7**	Pearson correlation	1	.690**	.914**
	Sig. (bilateral)		<.001	<.001
	N	408	406	406
**RAND8**	Pearson correlation	.690**	1	.924**
	Sig. (bilateral)	<.001		<.001
	N	406	407	406
**RAND_BP**	Pearson correlation	.914**	.924**	1
	Sig. (bilateral)	<.001	<.001	
	N	406	406	406

**. The correlation is significant at the 0.01 level (bilateral).

BP: Pain

Table. Item-item and item-total correlation matrices for RAND-36 "General health" dimension

		RAND1	RAND11a	RAND11b	RAND11c	RAND11d	RAND_GH
**RAND1**	Pearson correlation	1	.331**	.436**	.341**	.509**	.674**
	Sig. (bilateral)		<.001	<.001	<.001	<.001	<.001
	N	410	401	401	401	401	401
**RAND11a**	Pearson correlation	.331**	1	.339**	.350**	.294**	.667**
	Sig. (bilateral)	<.001		<.001	<.001	<.001	<.001
	N	401	404	404	404	404	401
**RAND11b**	Pearson correlation	.436**	.339**	1	.254**	.646**	.773**
	Sig. (bilateral)	<.001	<.001		<.001	<.001	<.001
	N	401	404	04	404	404	401
**RAND11c**	Pearson correlation	.341**	.350**	.254**	1	.368**	.643**
	Sig. (bilateral)	<.001	<.001	<.001		<.001	<.001
	N	401	404	404	404	404	401
**RAND11d**	Pearson correlation	.509**	.294**	.646**	.368**	1	.798**
	Sig. (bilateral)	<.001	<.001	<.001	<.001		<.001
	N	401	404	404	404	404	401
**RAND_GH**	Pearson correlation	.674**	.667**	.773**	.643**	.798**	1
	Sig. (bilateral)	<.001	<.001	<.001	<.001	<.001	
	N	401	401	401	401	401	401

**. The correlation is significant at the 0.01 level (bilateral).

GH: General health.

Table. Item-item and item-total correlation matrices for RAND-36 “Energy/fatigue” dimension

		RAND9a	RAND9e	RAND9g	RAND9i	RAND_VT
**RAND9a**	Pearson correlation	1	.706**	.402**	.445**	.812**
	Sig. (bilateral)		<.001	<.001	<.001	<.001
	N	403	401	402	402	400
**RAND9e**	Pearson correlation	.706**	1	.425**	.506**	.837**
	Sig. (bilateral)	<.001		<.001	<.001	<.001
	N	401	404	403	403	400
**RAND9g**	Pearson correlation	.402**	.425**	1	.693**	.765**
	Sig. (bilateral)	<.001	<.001		<.001	<.001
	N	402	403	405	405	400
**RAND9i**	Pearson correlation	.445**	.506**	.693**	1	.801**
	Sig. (bilateral)	<.001	<.001	<.001		<.001
	N	402	403	405	405	400
**RAND_VT**	Pearson correlation	.812**	.837**	.765**	.801**	1
	Sig. (bilateral)	<.001	<.001	<.001	<.001	
	N	400	400	400	400	400

**. The correlation is significant at the 0.01 level (bilateral).

VT: Energy/fatigue.

Table. Item-item and item-total correlation matrices for RAND-36 "Social functioning" dimension

		RAND6	RAND10	RAND_SF
**RAND6**	Pearson correlation	1	.630**	.904**
	Sig. (bilateral)		<.001	<.001
	N	407	398	398
**RAND10**	Pearson correlation	.630**	1	.902**
	Sig. (bilateral)	<.001		<.001
	N	398	400	398
**RAND_SF**	Pearson correlation	.904**	.902**	1
	Sig. (bilateral)	<.001	<.001	
	N	398	398	398

**. The correlation is significant at the 0.01 level (bilateral).

SF: Social functioning.

Table. Item-item and item-total correlation matrices for RAND-36 “Role limitations due to emotional problems” dimension

		RAND5a	RAND5b	RAND5c	RAND_RE
**RAND5a**	Pearson correlation	1	.836**	.724**	.922**
	Sig. (bilateral)		<.001	<.001	<.001
	N	403	402	401	401
**RAND5b**	Pearson correlation	.836**	1	.791**	.947**
	Sig. (bilateral)	<.001		<.001	<.001
	N	402	403	401	401
**RAND5c**	Pearson correlation	.724**	.791**	1	.906**
	Sig. (bilateral)	<.001	<.001		<.001
	N	401	401	401	401
**RAND_RE**	Pearson correlation	.922**	.947**	.906**	1
	Sig. (bilateral)	<.001	<.001	<.001	
	N	401	401	401	401

**. The correlation is significant at the 0.01 level (bilateral).

RE: Role limitations due to emotional problems.

Table. Item-item and item-total correlation matrices for RAND-36 “Emotional wellbeing” dimension.

		RAND9b	RAND9c	RAND9d	RAND9f	RAND9h	RAND_MH
**RAND9b**	Pearson correlation	1	.590**	.618**	.525**	.405**	.769**
	Sig. (bilateral)		<.001	<.001	<.001	<.001	<.001
	N	405	403	405	402	403	399
**RAND9c**	Pearson correlation	.590**	1	.580**	.754**	.559**	.856**
	Sig. (bilateral)	<.001		<.001	<.001	<.001	<.001
	N	403	403	403	400	402	399
**RAND9d**	Pearson correlation	.618**	.580**	1	.541**	.610**	.823**
	Sig. (bilateral)	<.001	<.001		<.001	<.001	<.001
	N	405	403	406	402	404	399
**RAND9f**	Pearson correlation	.525**	.754**	.541**	1	.595**	.839**
	Sig. (bilateral)	<.001	<.001	<.001		<.001	<.001
	N	402	400	402	402	400	399
**RAND9h**	Pearson correlation	.405**	.559**	.610**	.595**	1	.791**
	Sig. (bilateral)	<.001	<.001	<.001	<.001		<.001
	N	403	402	404	400	404	399
**RAND_MH**	Pearson correlation	.769**	.856**	.823**	.839**	.791**	1
	Sig. (bilateral)	<.001	<.001	<.001	<.001	<.001	
	N	399	399	399	399	399	399

**. The correlation is significant at the 0.01 level (bilateral).

MH: Emotional wellbeing.
